# A novel and efficient murine model for investigating tendon-to-bone healing

**DOI:** 10.1186/s13018-023-04496-9

**Published:** 2024-01-25

**Authors:** Baoyun Xu, Yunjiao Wang, Gang He, Kang-lai Tang, Lin Guo, Wan Chen

**Affiliations:** Department of Orthopaedic Surgery/Sports Medicine Center, Southwest Hospital, Army Medical University, Shapingba District, Chongqing, 400038 People’s Republic of China

**Keywords:** Tendon-to-bone healing, Mouse animal model, The Achilles tendon, Enthesis

## Abstract

**Background:**

Tendon-to-bone healing is a critical challenge in sports medicine, with its cellular and molecular mechanisms yet to be explored. An efficient murine model could significantly advance our understanding of this process. However, most existing murine animal models face limitations, including a propensity for bleeding, restricted operational space, and a steep learning curve. Thus, the need for a novel and efficient murine animal model to investigate the cellular and molecular mechanisms of tendon-to-bone healing is becoming increasingly evident.

**Methods:**

In our study, forty-four 9-week-old male C57/BL6 mice underwent transection and reattachment of the Achilles tendon insertion to investigate tendon-to-bone healing. At 2 and 4 weeks postoperatively, mice were killed for histological, Micro-CT, biomechanical, and real-time polymerase chain reaction tests.

**Results:**

Histological staining revealed that the original tissue structure was disrupted and replaced by a fibrovascular scar. Although glycosaminoglycan deposition was present in the cartilage area, the native structure had been destroyed. Biomechanical tests showed that the failure force constituted approximately 44.2% and 77.5% of that in intact tissues, and the ultimate tensile strength increased from 2 to 4 weeks postoperatively. Micro-CT imaging demonstrated a gradual healing process in the bone tunnel from 2 to 4 weeks postoperatively. The expression levels of ACAN, SOX9, Collagen I, and MMPs were detected, with all genes being overexpressed compared to the control group and maintaining high levels at 2 and 4 weeks postoperatively.

**Conclusions:**

Our results demonstrate that the healing process in our model is aligned with the natural healing process, suggesting the potential for creating a new, efficient, and reproducible mouse animal model to investigate the cellular and molecular mechanisms of tendon-to-bone healing.

**Graphical abstract:**

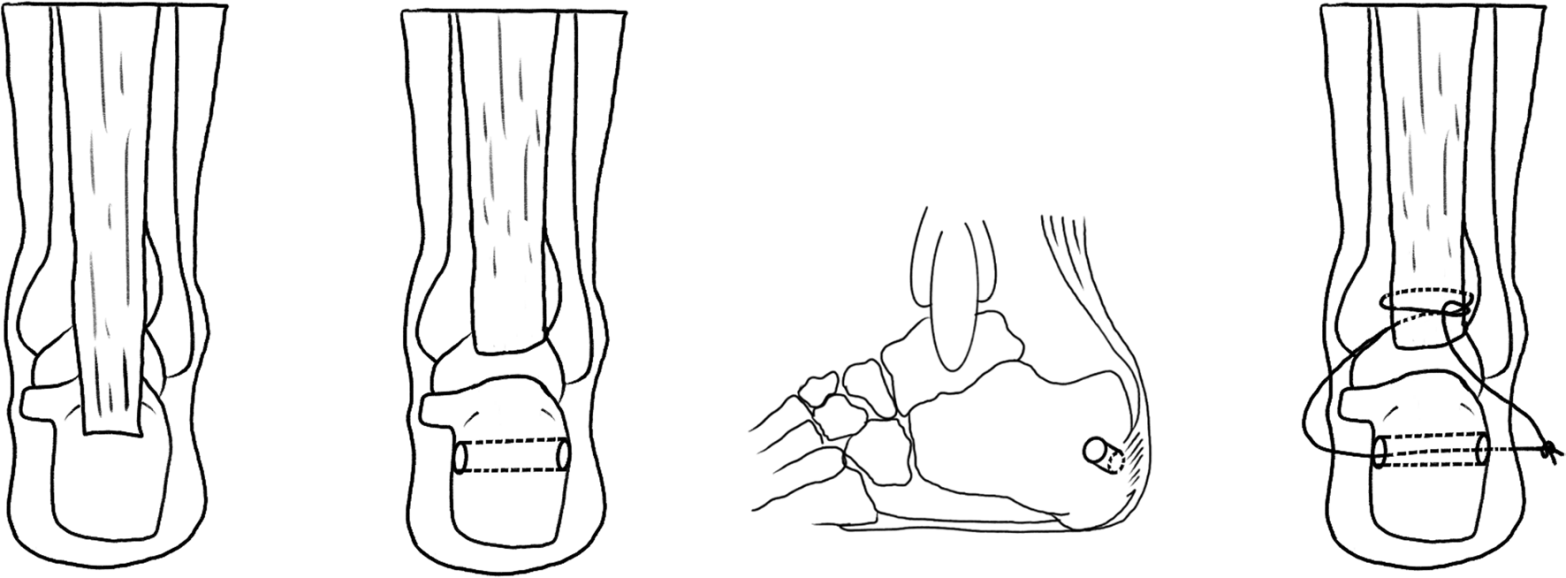

**Supplementary Information:**

The online version contains supplementary material available at 10.1186/s13018-023-04496-9.

## Background

Clinical research indicates that tendon and ligament injuries affect over 30 million individuals globally each year, and in the USA alone, more than 250,000 rotator cuff surgeries are performed annually [1, 2]. The primary goals of surgery are to alleviate pain and restore function; however, the high failure or recurrence rate presents a notable challenge. Studies [3–7] have shown that the recurrence rate for small-to-medium rotator cuff tears ranges from 20 to 40%, while large and chronic tears have a startling recurrence rate of up to 94%. A potential key reason is the formation of fibrovascular scar tissue at the repair site, replacing the original continuous four layers [8, 9]. This disordered structure demonstrates inferior mechanical properties compared to the native tissue [9]. Despite this, the molecular mechanisms underlying poor tendon-to-bone healing have received limited attention and remain largely unexplored. This significant scientific gap underscores the urgent need for further exploration and investigation to address this critical scientific issue.

Animal models play a crucial role for researchers studying tendon-to-bone healing (TBH). Large animals such as rabbits [10] and sheep [11] are frequently used in research to investigate TBH. Although large animal models are often employed to simulate human surgical procedures, their limited exploration in genetic and protein profiling restricts their use in molecular studies. In contrast, while small animals like mice may be considered too small for certain surgical procedures, they offer numerous indispensable advantages for studying mechanisms. These include easy access to transgenic mouse strains, significant anatomical resemblance to humans, substantial genetic similarity, cost-effectiveness in terms of acquisition, housing, and maintenance, and rapid growth rates. Therefore, despite their size constraints, mice serve as a valuable and versatile model system for exploring mechanisms in tendon-to-bone research.

Several articles [12–15] report on the use of mouse models for studying tendon-to-bone healing, predominantly focusing on rotator cuff repair. These models have notable limitations: the operational space in a mouse’s shoulder is extremely limited; the shoulder’s complex anatomy compounds this challenge; surgeries often lead to bleeding due to limited space; most procedures require a microscope, and authors typically provide brief descriptions of their methods, complicating replication efforts. These combined limitations make the mouse model impractical, necessitating a steep learning curve and resulting in a high failure rate. Therefore, developing a practical and efficient mouse model is essential to overcome these challenges and aid in investigating the molecular mechanisms of tendon-to-bone healing.

We have developed a novel and efficient mouse model that focuses on the enthesis between the Achilles tendon and the calcaneus, specifically for TBH research. We hypothesize that using this mouse model, known for its user-friendliness and productivity, can significantly enhance tendon-to-bone healing research. To validate the strength and reliability of our model, we conducted comprehensive evaluations, including biomechanical analyses, histological examinations, microcomputed tomography (Micro-CT) imaging, and real-time polymerase chain reaction (PCR) assessments.

## Methods

### Study design

**Fig. 1:**
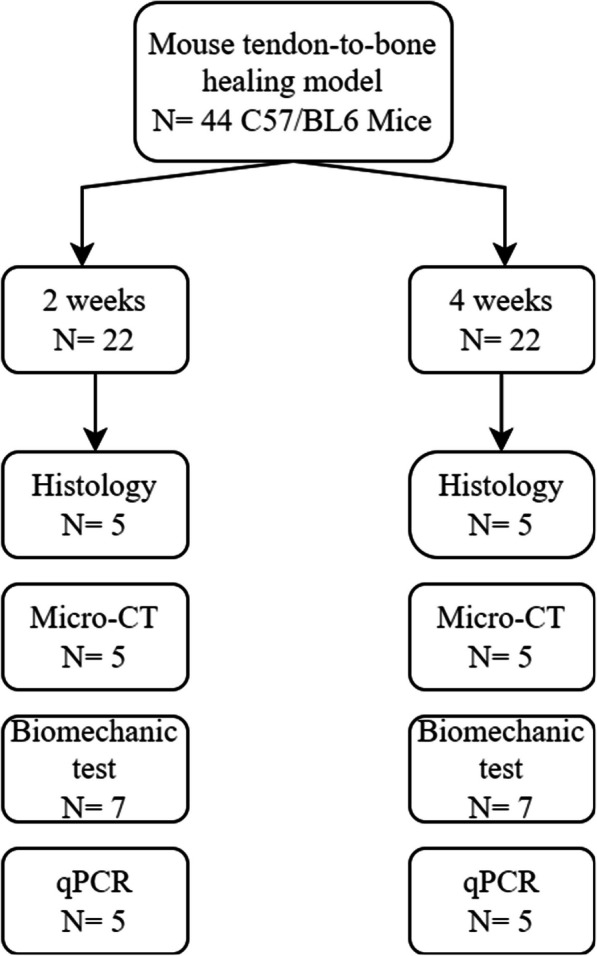
Experimental design

This study received approval from the Animal Ethics Committee of our university (approval number: AMUWEC20210782). We obtained forty-four 9-week-old male C57/BL6 mice (Hunan SJA Laboratory), designating their left hindfoot to the control group and their right hindfoot to the model group. In the model group, we applied novel surgical techniques, while the control group was left untreated to serve as a comparative baseline. At two and four weeks post-operation, the mice were killed for histological staining, Micro-CT scanning (Kontich, Belgium), biomechanical testing (TA Instruments, USA), and real-time PCR analysis (Fig. 1).

### Surgical procedures

**Fig. 2:**
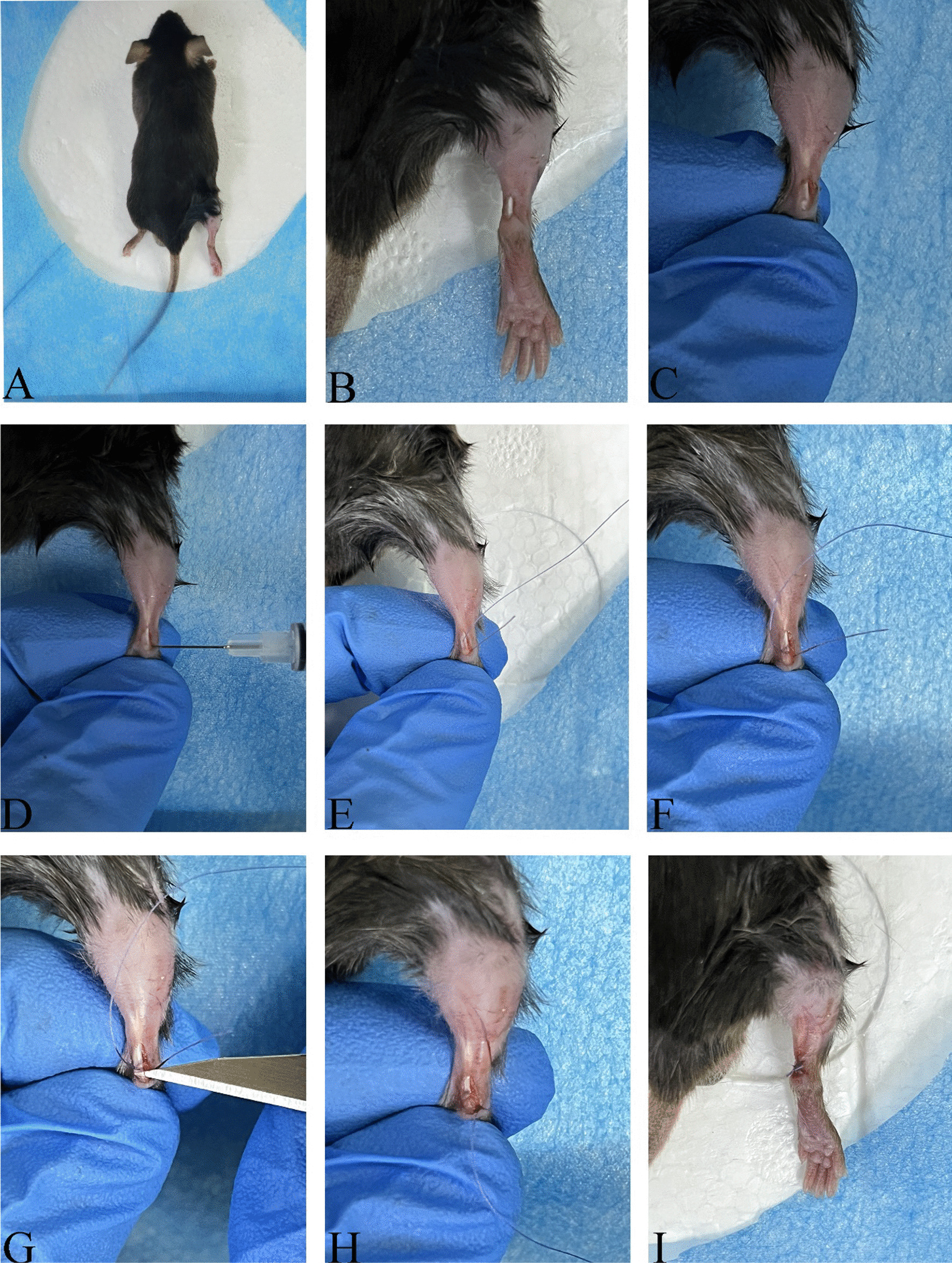
Surgical procedures: **A** The mouse is positioned in a prone position. **B** A 2 mm incision is made centrally, approximately 2 mm above the calcaneus. **C** The Achilles tendon and calcaneus are exposed. **D** A transverse calcaneus tunnel is perforated using a 30-G insulin needle. **E** A 6-0 absorbable suture is passed through the bone tunnel to suture the left part of the Achilles tendon, starting from the bottom and moving upwards. **F** The suture is looped around the Achilles tendon. **G** The Achilles tendon is incised at the insertion point. **H** The Achilles tendon is reattached to the calcaneus. **I** The skin incision is closed

**Fig. 3:**
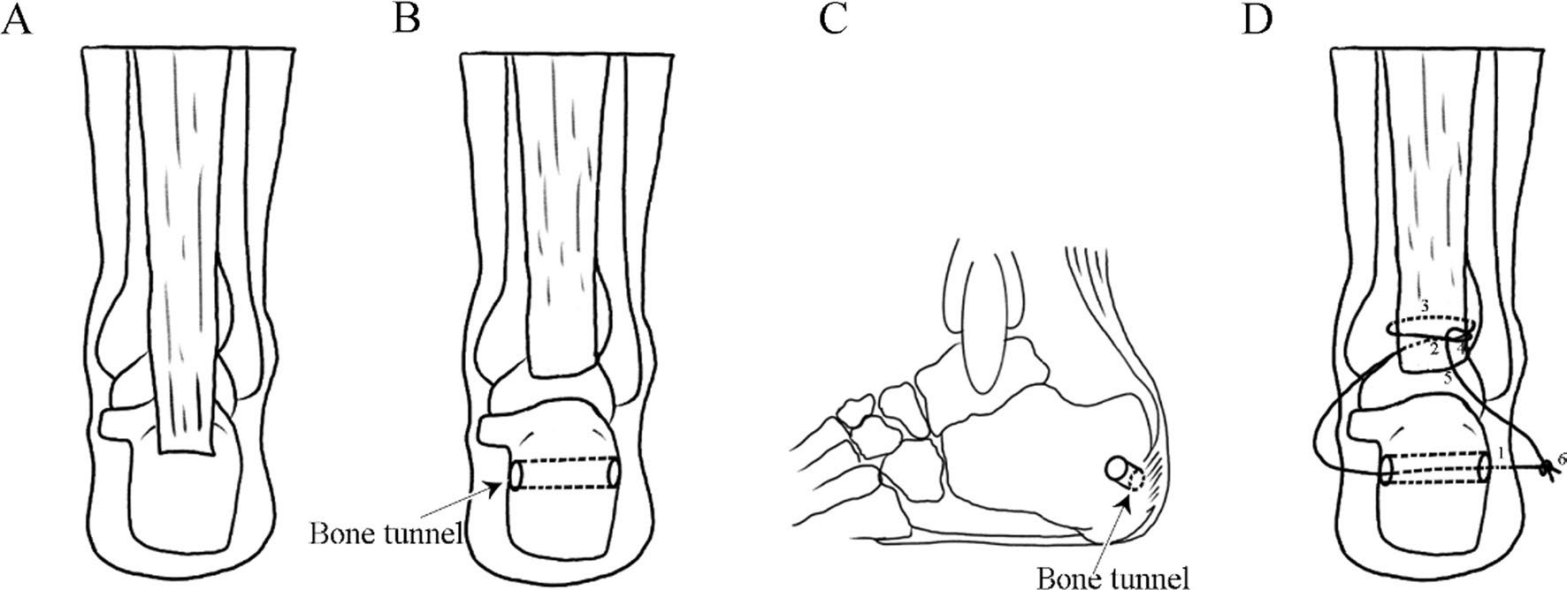
Graphical Abstract of Surgical Procedures: **A** Calcaneus and Achilles tendon; **B** and **C** Transverse bone tunnel in the calcaneus; **D** Tendon suturing techniques

The surgery was performed by a clinical doctor and a laboratory technician, under the supervision of a senior clinical doctor from the Sports Medicine Department. They refined and practiced the model techniques using cadaveric specimens collected from abolished mice (Refer to Figs. 2, 3, and Additional file 1: Video S1).

The mice were anesthetized with isoflurane and placed in a prone position on a foam surgical pad (Fig. 2A). After sterilization of the hindfoot, a 2 mm incision was made centrally, about 2 mm above the calcaneus (Fig. 2B). First, the tissue was dissected using blunt techniques to expose the Achilles tendon and calcaneus (Fig. 2C). Second, a transverse bone tunnel was created in the calcaneus with a 30 G insulin needle (Fig. 2D). Next, a 6-0 absorbable suture was threaded through the bone tunnel and the Achilles tendon in an oblique pattern from lower left to upper right, looped around the tendon toward the lower right, then passed obliquely from lower right to upper left, and secured (Fig. 2E and F). Third, the Achilles tendon was carefully incised near its insertion with a No.11 blade, and the cartilage at the tendon-to-bone interface (TBI) was gently excised (Fig. 2G). The suture ends were then knotted and tightened (Fig. 2H). Finally, the incision was closed with 1–2 interrupted sutures (Fig. 2I). Post-surgery, the mice had unrestricted movement in their cages, with free access to water and food throughout the experiment.

### Morphological and histological observation

At 2 and 4 weeks post-surgery, five mice were killed for specimen collection. The skin was removed, and the calcaneus along with a portion of the Achilles tendon was harvested. These specimens were preserved in 4% paraformaldehyde for 24 h. Subsequently, they underwent decalcification in an EDTA solution for 72 h at 37 degrees Celsius in a thermostatic shaker. The specimens were then embedded in paraffin and sectioned sagittally. Hematoxylin and eosin (H&E), Sirius red, and safranin O fast green staining were conducted to examine the tendon-to-bone interface. H&E staining assessed the overall condition, including new tissue formation and cell morphology. Sirius red staining evaluated tendon maturation, and safranin O fast green staining detected glycosaminoglycan and cartilage formation. Additionally, we used a modified tendon maturity assessment system [16], comprising eight items for a total score of 32, to evaluate tendon maturity, with a higher score indicating more advanced tendon-to-bone healing.

### Micro-CT scanning

Two and four weeks post-surgery, three mice were killed for specimen collection to study bone formation within the bone tunnel. The samples were analyzed using Micro-CT with the Bruker MicroCT Skyscan 1272 system (Kontich, Belgium), which featured an isotropic voxel size of 10.0 µm to image the entire calcaneus. Scans were performed in 4% paraformaldehyde, utilizing an X-ray tube potential of 60 kV, X-ray intensity of 166 µA, and an exposure time of 1700 ms. Image reconstruction was conducted using Nrecon software (Ver. 1.6.10), and three-dimensional (3D) images were generated from two-dimensional (2D) scans using CTvox software (Ver. 3.0.0), based on distance transformation of the grayscale original images.

### Biomechanical test

Seven mice were killed, and only the entire feet and tendons were harvested at both the 2-week and 4-week postoperative time points. Ultimate tensile strength (MPa), stiffness (N/mm), and failure force were assessed using ElectroForce Mechanical Testing Instruments (TA Instruments, USA). To enhance fixation friction, the specimens were wrapped in gauze before being secured in the tensile testing machine. Following calibration, a preload of 0.05 N was applied for 1 min, and then, the samples were stretched at a rate of 0.03 mm/s. Data associated with terminal tendon slips and fractures not related to the tendon-to-bone insertion sites were excluded from our analysis.

### Real-time PCR

At each postoperative time point, five mice were killed, and we collected samples from the tendon-to-bone interfaces, which included small portions of both the bone and tendon. These specimens were then sectioned, and total RNA was extracted using TRIzol (Thermo Fisher Scientific, USA). The concentration of the extracted RNA was determined by spectrophotometry and normalized against the expression of the housekeeping gene GAPDH. The primer sequences for genes ACAN, COL1a1, SOX9, MMP3, MMP13, MMP14, and GAPDH are provided below (See Table 1).

**Table 1:** Primer sequences

Gene	Forward	Reverse
Col1a1	AGC ACG TCT GGT TTG GAG AG	GAC ATT AGG CGC AGG AAG GT
ACAN	TTG ACG AGT GCC TCT CAA GC	GCT CCT GGT CGA TCT CAC AC
Sox9	GTG CAA GCT GGC AAA GTT GA	TGC TCA GTT CAC CGA TGT CC
MMP3	GTT CTG GGC TAT ACG AGG GC	TTC TTC ACG GTT GCA GGG AG
MMP13	GGC TGG AAC CAC ATG GAA GA	ATG GAC CCC ATG TT GCT GT
MMP14	TCA CCC CAG CAT TGC TTC AT	CAC ACA CCG AGC TGT GAG AT
GAPDH	GGT TGT CTC CTG CGA CTT CA	TGG TCC AGG GTT TCT TAC TCC

### Statistical analysis

Data of two groups were presented as mean ± SD. After Shapiro–Wilk normality test, two groups were compared with unpaired t tests, and differences were considered significant when *P* < 0.05. The statistical analysis was performed using R software (Version 4.3.0).

## Results

### Operative time

The average skin-to-skin time for beginners was approximately 10 min. However, this time decreased to about 6 min after they practiced on several cadaveric mice. Notably, there were no instances of significant bleeding during the surgical procedures.

### Morphological and histological observation

**Fig. 4:**
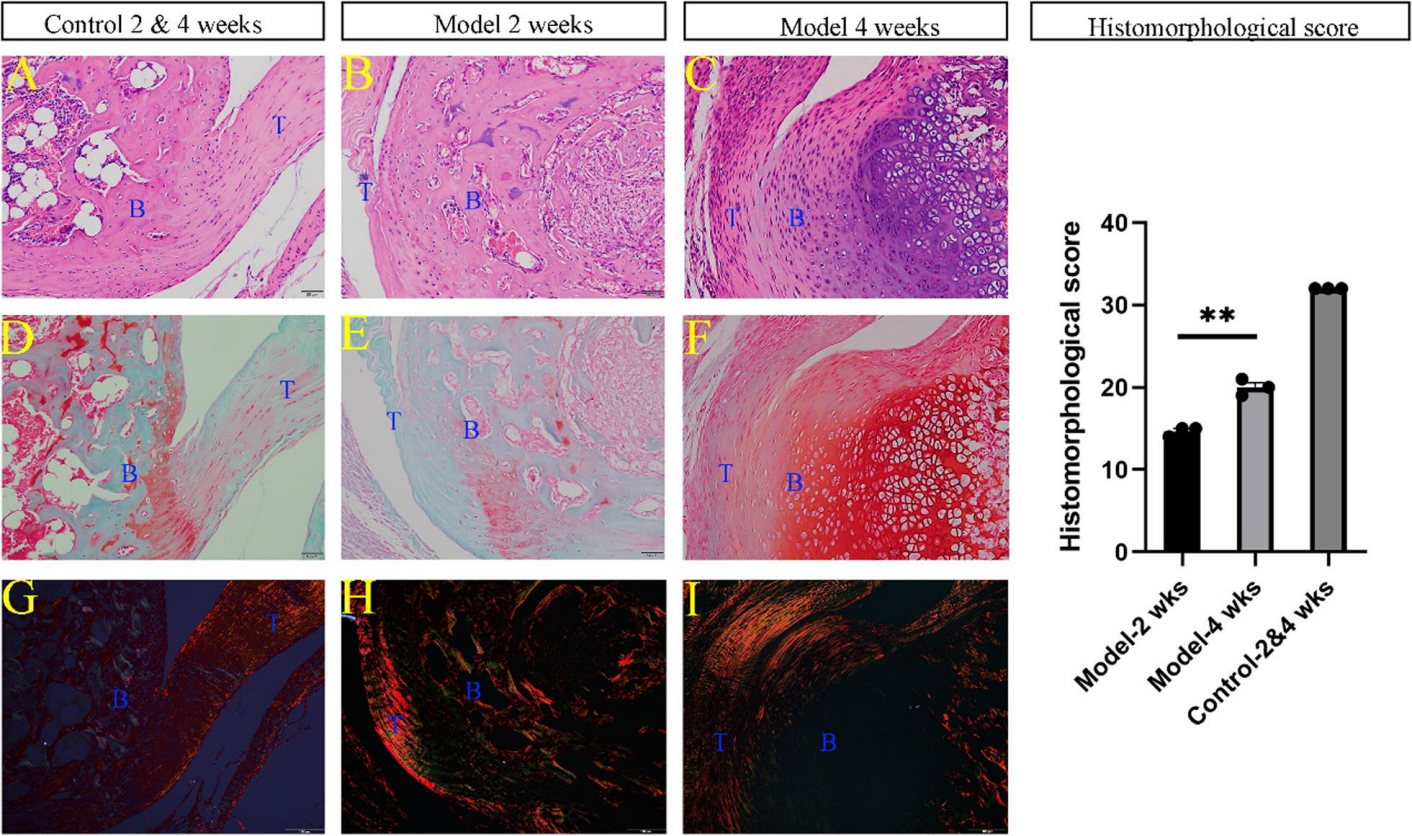
Histological staining of tendon-to-bone interface (20X). **A**, **D**, **G** H&E staining, safranin O fast green staining and Sirius red staining of control group, respectively. **B**, **E**, **H** H&E staining, safranin O fast green staining and Sirius red staining of model group at 2 weeks postoperatively, respectively. **C**, **F**, **I** H&E staining, safranin O fast green staining and Sirius red staining of model group at 4 weeks postoperatively, respectively. B, bone (calcaneus), T tendon (the Achilles tendon)

After harvesting, we observed thickening of the Achilles tendon, structural disorder, and a loss of original luster and toughness compared to native tissue at both 2 and 4 weeks postoperatively. At 2 weeks, H&E staining revealed thickening of the Achilles tendon, disordered fibers, and incomplete reattachment to the calcaneus. This was coupled with a disruption of the original four layers and the formation of a fibrovascular scar at the tendon-to-bone interface. At 4 weeks, H&E staining indicated complete reattachment of the Achilles tendon to the calcaneus, yet the tendon and tendon-to-bone interface structure remained disordered. Chondrocytes in the tendon-to-bone interface were noted, but they lacked a smooth transition structure. Similarly, safranin O fast green staining showed glycosaminoglycan deposition at this site, but the original non-calcified and calcified structures were absent. Observations under a polarization microscope of Sirius red-stained slices revealed aligned tendons rich in type I collagen (red) in the control group, in contrast to the model group, where the tendon was disrupted and replaced by a fibrovascular scar (green). The average tendon maturity score in the model group was 14.67 ± 0.47 at two weeks postoperatively, increasing to 20 ± 0.82 at four weeks, suggesting an enhancement in the tendon-to-bone connection (Fig. 4).

### Micro-CT scanning

**Fig. 5:**
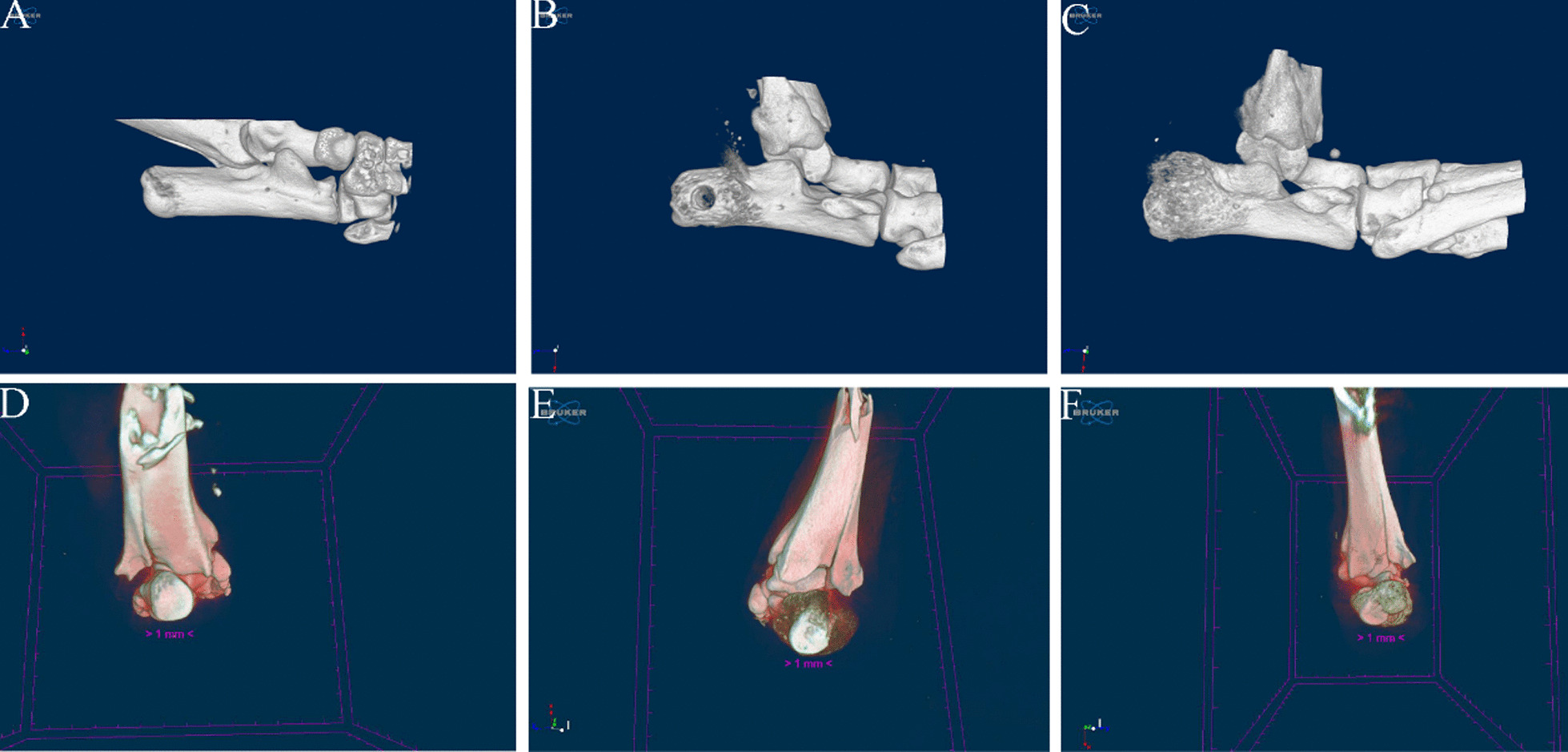
3D reconstruction of calcaneus. **A** Calcaneus of control group. **B** Calcaneus of model group at 2 weeks postoperatively. **C** Calcaneus of model group at 4 weeks postoperatively. **D** Interface of control group. **E** Interface of model group at 2 weeks postoperatively. **F** Interface of model group at 4 weeks postoperatively

3D image reconstruction allowed us to observe the persistence of the bone tunnel at two weeks postoperatively. By four weeks postoperatively, significant healing and closure of the bone tunnel were evident. Additionally, we noted an increase in bone mass at the tendon-bone interface over time, accompanied by a more complete structural formation (Fig. 5).

### Biomechanical test

**Fig. 6:**
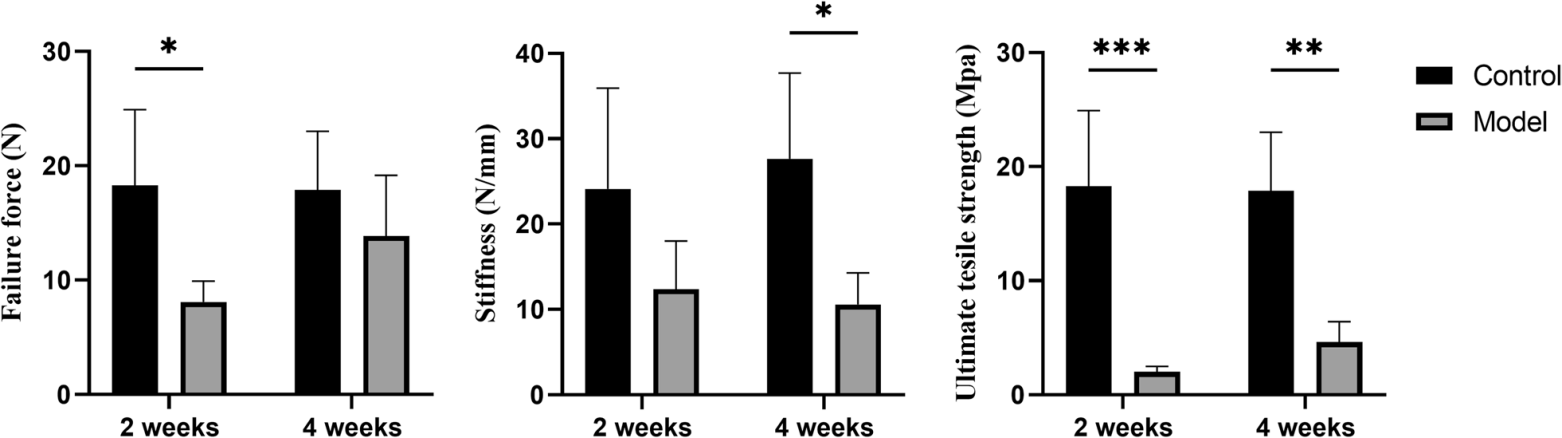
Statistical data of biomechanical results between control and model group. (*P < 0.05, **P < 0.01, ***P < 0.001)

The failure force of the experimental group at 2 and 4 weeks post-operation was 8.08 ± 1.82 and 13.85 ± 5.32, respectively (*P* = 0.03), accounting for approximately 44.2% and 77.5% of that in intact tissues. The stiffness of the experimental group at 2 and 4 weeks post-operation was 12.37 ± 5.66 and 10.58 ± 3.7, respectively, with no significant difference between the two time points (P = 0.53). The ultimate tensile strength of the experimental group at 2 and 4 weeks post-operation was 2.02 ± 0.46 and 4.62 ± 1.78, respectively (*P* = 0.005). These biomechanical test results indicate that the biomechanical strength of the model group increases over time, correlating with the normal healing process (Table 2 and Fig. 6).

**Table 2:** Biomechanical results for tendon-to-bone healing model

	Failure force (N)	Stiffness (N/mm)	Ultimate tensile strengths (Mpa)
Intact 2 weeks	18.29 ± 6.63	24.13 ± 11.77	18.29 ± 6.63
Intact 4 weeks	17.88 ± 5.12	27.65 ± 10.04	17.88 ± 5.12
Post-operation 2 weeks	8.08 ± 1.82	12.37 ± 5.66	2.02 ± 0.46
Post-operation 4 weeks	13.85 ± 5.32	10.58 ± 3.7	4.62 ± 1.78

### Real-time PCR

**Fig. 7:**
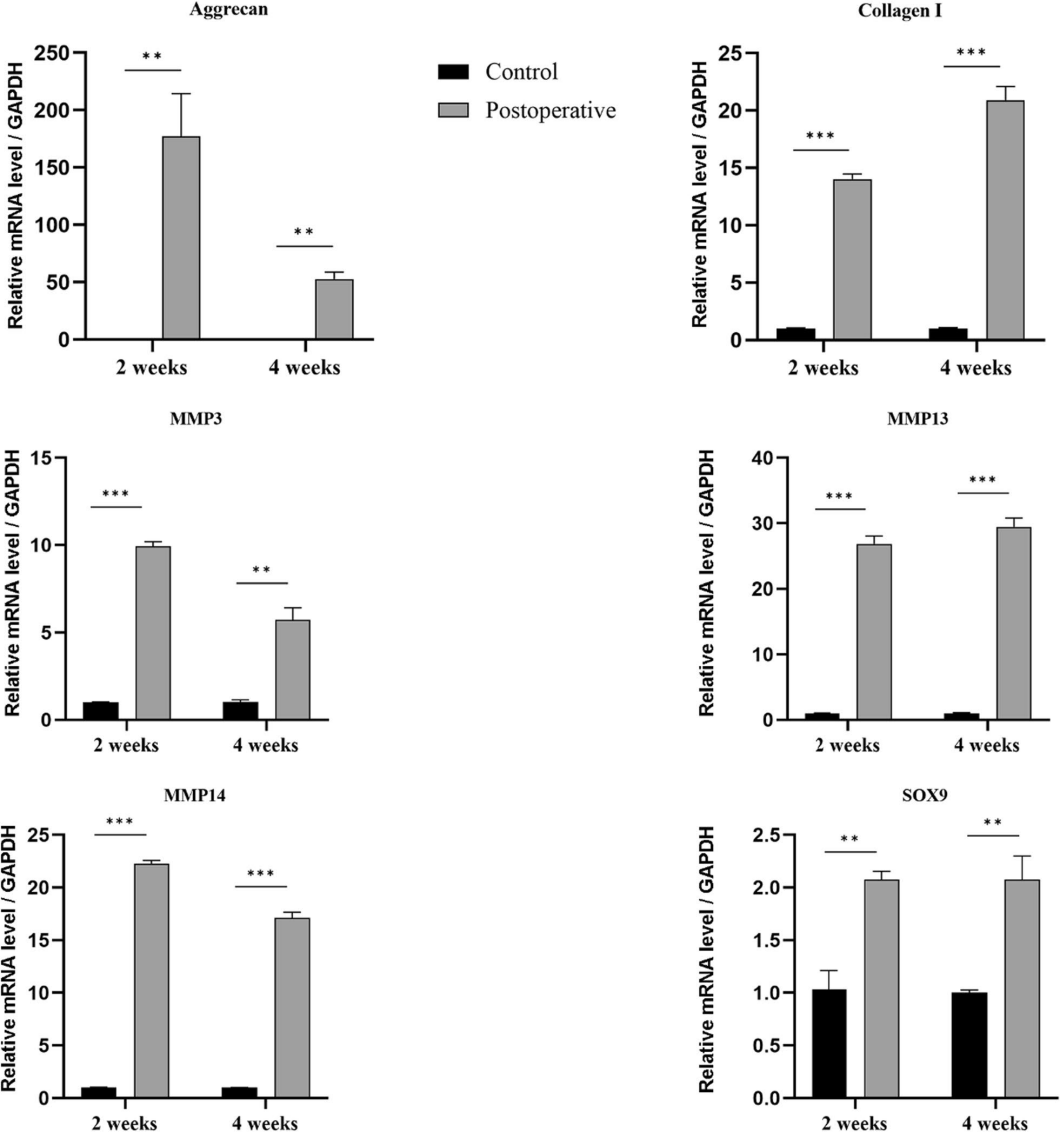
Gene analysis of tendon-to-bone interface. (**P < 0.01, ***P < 0.001)

According to the data, key catabolic indicators, including MMP3 and MMP14, peaked at 2 weeks postoperatively. Although MMP13 exhibited a slight increase, there was no significant difference between the 2-week and 4-week time points. In contrast, anabolic indicators like SOX9 and Collagen I reached their peak in the fourth week. However, ACAN demonstrated a decreasing trend by the fourth week. These results suggest that most tissue inflammation occurs during the second week post-surgery, while tissue remodeling and maturation predominantly occur in the fourth week (Fig. 7).

## Discussion

From the histological and biomechanical test results, we noted a progressively stronger bond forming between the Achilles tendon and the calcaneus in our animal model, characterized by the absence of a discernible gap between the tendon and bone. The maturity of the tendon-to-bone insertion increased over time. Moreover, our PCR tests revealed a sequential healing process that progresses from inflammation to tissue remodeling, suggesting that our new TBH model effectively mirrors the natural tendon-to-bone healing process.

As previously mentioned, current murine models focusing on tendon-to-bone healing have certain limitations. One study [14] reported a mouse rotator cuff repair model, including the tendon-to-bone interface; we tested these methods, finding them effective yet demanding a significant learning curve. Furthermore, these procedures are prone to bleeding and often require surgery under a microscope. Consequently, we developed a model centered on the Achilles tendon insertion, offering several advantages: (1) Easier exposure; (2) Reduced risk of bleeding due to the absence of major blood vessels, unlike in the shoulder region; (3) Ample space for surgical maneuvers. This approach streamlines the development of an animal model and facilitates a deeper investigation into the mechanisms of tendon-to-bone healing.

Our morphological and histological tests indicate that following reattachment, the insertion of the Achilles tendon undergoes a gradual process of inflammation and tissue remodeling. Our cartilage staining reveals the deposition of glycosaminoglycans in the area of the tendon-to-bone interface. The histomorphological score yielded results consistent with Ide’s study [16]. However, the original four transitional structures have been disrupted and replaced by a fibrovascular scar. Generally, studies [17, 18] have reported the tendon-to-bone healing process involves inflammation, proliferation and remodeling; furthermore, the original tendon-to-bone insertion is typically replaced by a fibrovascular scar. Our results correspond with these natural healing processes, which further enhance the robustness and value of this animal model.

Biomechanical tests demonstrate an increase in the failure force and ultimate tensile strength from 2 to 4 weeks postoperatively. And failure force accounts for approximately 44.2% and 77.5% of intact tissues, which is consistent with the findings of the mice rotator cuff repair model (34% and 75%) reported by Lebaschi et al. [14]. Although the stiffness has slightly decreased at the fourth week, there is no statistically significant difference between the 2-week and 4-week postoperative periods.

Several studies [19, 20] have identified critical factors involved in tendon-to-bone healing, including SOX9, Collagen I, ACAN, and MMPs. SOX9 plays a vital role in chondrogenesis and chondrocyte differentiation. Collagen I and ACAN are key matrix genes, while MMPs are essential for tissue remodeling. Research [21] has shown that inhibiting MMP13 affects the early stages of tendon-to-bone healing. In our study, these genes were overexpressed compared to the control group and maintained high expression levels, aligning with the findings reported by Lebaschi [14] and Oshiro et al. [22]

We conducted this experiment and provided a detailed description of our surgical procedures. It is well known that the small size of mice poses limitations on replicating surgeries performed on humans. Many mouse models, such as the osteoarthritis model and the rotator cuff repair model, require surgical procedures under a microscope. The biggest advantage of our new murine animal model is that surgeries can be performed without microscope. It is noteworthy that once researchers become proficient in the surgical procedures, they can complete the surgery in less than 6 min, highlighting the efficiency and proficiency achieved through experience. Furthermore, we demonstrated that our model’s tendon-to-bone healing process aligns with both the natural healing process and the rotator cuff repair model in terms of biomechanical and histological results. Therefore, we believe that our new animal model can assist researchers in addressing issues related to tendon-to-bone healing and inform clinical practices. However, limitations exist due to the small size of the mouse tendon and calcaneus, which may not perfectly replicate human surgical procedures. Further studies are needed to better emulate human surgeries in a mouse model.

## Conclusion

The model successfully replicated key aspects of tendon-to-bone interface healing, including tissue thickening, structural changes, scar formation, and biomechanical properties. This suggests that the developed murine animal model offers a new, efficient, and reproducible platform for studying the mechanism of tendon-to-bone healing. This model would be valuable for further investigations into the molecular and cellular mechanisms underlying tendon-to-bone healing.

### Supplementary Information

**Additional file 1:** Video S1.

## Data Availability

The data supporting the findings of this study are available within the article and its supplementary information files.

## Abbreviations

TBH: Tendon-to-bone healing
Micro-CT: Microcomputed tomography
PCR: Real-time polymerase chain reaction
TBI: Tendon-to-bone interface
H&E: Hematoxylin and eosin
3D: Three-dimensional
